# Genome-Wide Identification, Phylogeny, and Expression Analysis of ARF Genes Involved in Vegetative Organs Development in Switchgrass

**DOI:** 10.1155/2018/7658910

**Published:** 2018-04-29

**Authors:** Jianli Wang, Zhenying Wu, Zhongbao Shen, Zetao Bai, Peng Zhong, Lichao Ma, Duofeng Pan, Ruibo Zhang, Daoming Li, Hailing Zhang, Chunxiang Fu, Guiqing Han, Changhong Guo

**Affiliations:** ^1^College of Life Science and Technology of Harbin Normal University, Harbin 150080, China; ^2^Key Laboratory of Biofuels, Shandong Provincial Key Laboratory of Energy Genetics, Qingdao Institute of Bioenergy and Bioprocess Technology, Chinese Academy of Sciences, Qingdao 266101, China; ^3^Grass and Science Institute of Heilongjiang Academy of Agricultural Sciences, Harbin, Heilongjiang 150086, China; ^4^Rural Energy Research Institute of Heilongjiang Academy of Agricultural Sciences, Harbin, Heilongjiang 150086, China

## Abstract

Auxin response factors (ARFs) have been reported to play vital roles during plant growth and development. In order to reveal specific functions related to vegetative organs in grasses, an in-depth study of the ARF gene family was carried out in switchgrass (*Panicum virgatum* L.), a warm-season C4 perennial grass that is mostly used as bioenergy and animal feedstock. A total of 47 putative ARF genes (*PvARFs*) were identified in the switchgrass genome (2n = 4x = 36), 42 of which were anchored to the seven pairs of chromosomes and found to be unevenly distributed. Sixteen *PvARFs* were predicted to be potential targets of small RNAs (microRNA160 and 167). Phylogenetically speaking, PvARFs were divided into seven distinct subgroups based on the phylogeny, exon/intron arrangement, and conserved motif distribution. Moreover, 15 pairs of *PvARFs* have different temporal-spatial expression profiles in vegetative organs (2nd, 3rd, and 4th internode and leaves), which implies that different *PvARFs* have specific functions in switchgrass growth and development. In addition, at least 14 pairs of *PvARFs* respond to naphthylacetic acid (NAA) treatment, which might be helpful for us to study on auxin response in switchgrass. The comprehensive analysis, described here, will facilitate the future functional analysis of ARF genes in grasses.

## 1. Introduction

Auxin, an essential plant hormone, plays vital roles in various aspects of plant growth and development, such as embryogenesis, organogenesis, tropic growth, shoot elongation, root architecture, flower and fruit development, tissue and organ patterning, and vascular development [1–9]. Most of these processes are controlled by auxin response genes, which are regulated at transcriptional level by *cis*-acting DNA elements in their promoter regions, including the auxin response element (AuxRE, TGTCTC), core of auxin response region (AuxRR-core, GGTCCAT), and TGA-element (AACGAC). Of these, AuxREs are reported to be specifically bound and regulated by a class of transcription factors, called auxin response factors (ARFs) [10, 11]. ARF proteins generally contain a DNA-binding domain (DBD) in the amino (N)-terminal region, a central region that functions as an activation domain (AD) or a repression domain (RD) [12, 13], and a carboxyl (C)-terminal dimerization domain (CTD), which is a protein-protein interaction domain that mediates ARF homo- and heterodimerization and also the heterodimerization of ARF and Aux/IAA proteins, another category of auxin response regulators [12–16].

Because of their important roles in auxin signaling pathways, which are indispensable to plant growth and development, ARF gene families have been studied in many plant species. For example, there are 23 ARF transcription factors in *Arabidopsis* (*Arabidopsis thaliana*) [17], 25 in rice (*Oryza sativa*) [18], 39 in poplar (*Populus trichocarpa*) [19], 24 in *Medicago truncatula* [20], and 36 in maize (*Zea mays*) [21]. In previous studies, ARF proteins were split into three clades (clades A, B, and C) based on phylogenic relationships, which could be traced back to the origin of land plants [22]. In particular, phylogenetic analysis of the ARF gene family in many species has been widely reported. Arabidopsis ARFs were divided into four subgroups, which is in accordance with the phylogenetic classifications of ARFs in rice [18], banana (*Musa acuminata* L.) [23] and *Salvia miltiorrhiza* [24]. Maize and poplar ARFs are classified to six subgroups [25], whereas *Medicago* ARFs were divided into eight subgroups [20]. In general, the wide variety of ARF phylogenetic grouping patterns are based on the diversification of its gene structure and motif locations, which may be the result of gene truncation or alternative splicing [22].

Biochemical and genetic analyses have established the crucial roles of ARF genes in plant growth and development. In *Arabidopsis thaliana*, AtARF2 regulates floral organ abscission, leaf senescence, and seed size and weight [26–28]. AtARF5 affects vascular development and early embryo formation [29]. AtARF8 controls the uncoupling of fruit development from pollination and fertilization, and loss-of-function mutations in these genes result in seedless fruit [30]. AtARF7 and AtARF19 redundantly regulate auxin-mediated lateral root development [31]. In rice, OsARF1 is required for vegetative and reproductive development [32]. OsARF16 is essential for iron and phosphate deficiency responses in rice [33]. In addition, some ARF genes are involved in the response to abiotic stresses, such as drought, salt, or cold stress [34, 35]. Taken together, these studies have shown that the ARF gene family function in multiple signal transduction pathways to regulate multiple aspects of plant growth and development.

Switchgrass (*Panicum virgatum* L.) is a warm-season C4 perennial grass used as a bioenergy and animal feedstock [36, 37]. To avoid competing with food crops for arable fields, a large proportion of switchgrass fields will be located on marginal lands where various abiotic stresses, such as salt, drought, and extreme temperatures. The genome sequence of switchgrass has been published recently [38] and provides a powerful resource to identify ARF gene family members. Considering the value of switchgrass as a bioenergy and animal feedstock, we mainly focused on vegetative organs in this study.

Here, we identified 47 switchgrass ARF genes and comprehensively characterized the physical location, conserved motif architecture, and expression profile of the *PvARFs*. We also subdivided these 47 PvARF genes based on phylogenetic relationships based on the well-studied ARF genes in other species. To determine which ARF genes potentially work on different developmental processes, the temporal-spatial expression pattern in vegetative organs (2nd, 3rd, and 4th internode and leaves) and expression response to auxin treatment in seedlings were determined by real-time PCR (qRT-PCR). Our works provide preliminary information about ARF genes in switchgrass and lays the foundation for the further elucidation of the biological roles of ARF genes in grasses.

## 2. Materials and Methods

### 2.1. Plant Materials and Treatments

A widely used and highly productive lowland-type switchgrass cultivar, Alamo, was grown in the greenhouse at 28 ± 1°C with 16 h lighting, followed by 8 h darkness. Switchgrass development in our greenhouse was divided into three vegetative stages (V1, V2, and V3), five elongation stages (E1, E2, E3, E4, and E5), and three reproductive stages (R1, R2, and R3). Six different tissues, including the second internode (I2), the third internode (I3), the fourth internode (I4), the second leaf (L2), the third leaf (L3), and the fourth leaf (L4), were collected at the R2 stage [39].

For auxin treatments, plantlets grown in tissue culture until 20 days after rooting were incubated for 1, 2, and 3 h in hormone-supplemented 5 *μ*M naphthylacetic acid (NAA) medium [18]. Control plants were grown in hormone-free medium. Whole seedlings were sampled from NAA-treated and control plants at the same time points. All experiments included three biological replicates. All of the samples were stored at −80°C.

### 2.2. Sequence Retrieval and Identification

The conserved ARF domain based on the Hidden Markov Model (HMM) (Pfam06507) was obtained from the Pfam protein family database (http://pfam.sanger.ac.uk/) and used as a query to search against the switchgrass genome database in Phytozome v11 (http://www.phytozome.net/). Sequences were selected for further analysis if the E value was less than 1e-10. Several coding sequences (CDS) were corrected based on the switchgrass unique transcript sequence database [38]. Peptide length, molecular weight, and isoelectric point of each PvARF were calculated using the online ExPASy program (http://www.expasy.org/).

### 2.3. Phylogenetic Analysis

The putative PvARF proteins from another fifteen species were used to construct a phylogenetic tree. ARF protein sequences were obtained from the public genome database Phytozome. The BlastP program was used to identify putative ARF proteins from the genomic databases of well-sequenced species, including *Arabidopsis*, sweet orange (*Citrus sinensis*), Chinese cabbage (*Brassica rapa*), poplar, *Medicago* (*Medicago truncatula*), cotton (*Gossypium raimondii*), *Grandis* (*Eucalyptus grandis*), soybean, tomato (*Solanum lycopersicum*), grape (*Vitis vinifera*), maize, rice, foxtail millet (*Setaria italica*), sorghum (*Sorghum vulgare*), and *Brachypodium distachyon*. Multiple sequence alignments of the full-length ARF sequences were performed using Clustal X1.83, and the edges of the alignments were manually trimmed. An unrooted neighbor-joining (bootstrap value = 1000) tree was constructed using MEGA5 and then manually improved by online program EvolView (http://www.evolgenius.info/evolview/).

### 2.4. Chromosomal Locations of PvARF Genes

The lowland switchgrass cultivars are allotetraploid (2n = 4x = 36) and consist of two highly homologous subgenomes, designated as Chr.a and Chr.b [40]. Specific physical locations of each PvARF were obtained from the Phytozome database. Chromosome locations were then determined using MapChart 2.2 based on the genetic linkage map [41, 42]. Tandem gene duplicates were defined as paralogous genes located within 50 kb and separated by fewer than five nonhomologous spacer genes [43].

### 2.5. Gene Structure, Conserved Motif, and *Cis*-Acting DNA Element Analysis

A comparison of each CDS with the corresponding genomic DNA sequence was made to determine the positions and numbers of introns and exons of each PvARF gene using the Gene Structure Display Server (http://gsds.cbi.pku.edu.cn/). Conserved motifs were analyzed using the MEME program (http://meme.nbcr.net/meme/cgi-bin/meme.cgi). Putative microRNA target sites in PvARFs were identified using the miRanda online software (http://cbio.mskcc.org/microrna_data/manual.html). *Cis*-acting DNA elements were analyzed using the PLACE online program (https://sogo.dna.affrc.go.jp/) [44]. *Ka*/*Ks* calculation was analyzed by PAL2NAL [45].

### 2.6. Gene Expression Analysis by qRT-PCR

Probesets of PvARF genes were retrieved from public database of switchgrass (https://switchgrassgenomics.noble.org/). qRT-PCR was performed to analyze the transcript abundance of PvARFs in different switchgrass tissue samples. Plant tissue samples were ground in liquid nitrogen using a mortar and pestle. Total RNA was isolated using the TRIZOL reagent according to the manufacturer's supplied protocol (Transgen, China) and subjected to reverse transcription with Superscript PrimeScript™ RT reagent Kit (TaKaRa, China) after treatment with TURBO DNase I (TaKaRa, China). The qRT-PCR primers were designed using Primer Premier 5 (Table S1), and their specificity was verified by PCR. qRT-PCR analysis was conducted in triplicate using SYBR® Premix Ex Taq™ II (TaKaRa, Japan), with PvUBQ as a reference gene, with a Light Cycler 480 real-time PCR system (Roche, Switzerland). The qRT-PCR reactions and data analyses were performed according to previously published methods [46].

## 3. Results

### 3.1. Identification and Chromosomal Localization of Switchgrass ARFs

To identify ARF proteins in switchgrass, the Hidden Markov Model (HMM) profile of the conserved ARF domain (Pfam06507) was used as a query to search against the publicly available switchgrass genome database (Phytozome v11) by BlastP and tBlastN program. A total of 47 putative ARF proteins were found, and Pfam analysis confirmed that all of these proteins contain the ARF domain. The putative candidates were designated as PvARF1 to PvARF47, based on the alignments of predicted amino acid sequences. The predicted PvARF proteins ranged from 243 (PvARF34) to 1182 (PvARF13) amino acids (aa) in length and from 27.4 kDa to 130.9 kDa in molecular weight (Table 1). The isoelectric points (*pI*) ranged from 5.33 (PvARF8) to 9.52 (PvARF25) (Table 1), suggesting that different PvARFs might have roles in specific subcellular environments.

**Figure 1:**
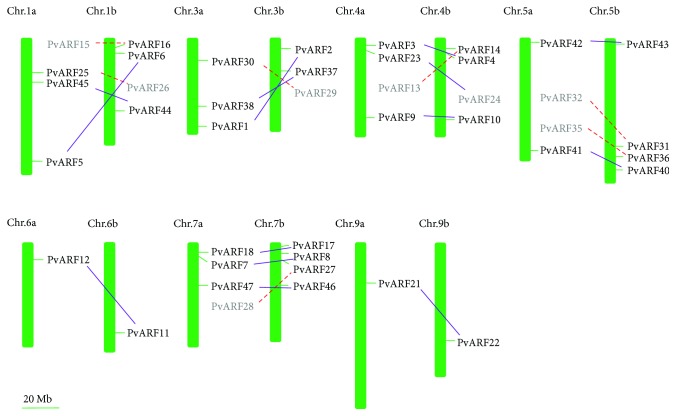
Chromosomal distribution of ARF genes in switchgrass. Distribution on the chromosomes (vertical bar) indicate the position of centromeres. The chromosome numbers (except for Chromosome 2a, 2b, 8a, and 8b) are indicated at the top of each chromosome image. The purple solid lines show the gene pairs, and the red dotted lines represent the putative gene pairs according to the sequence similarity.

To examine the chromosomal distribution of *PvARFs*, the physical locations of the *PvARFs* on chromosomes (Chrs) were obtained through BlastN searches against the switchgrass genome database in Phytozome. Due to the allotetraploidization of switchgrass (2n = 4x = 36), the PvARF genes exist as paralogous gene pairs in the genome with only one exception, *PvARF39*, which might be lost in the evolutionary process. Of the 47 *PvARFs*, 42 were putatively anchored onto seven of the nine switchgrass chromosomes (Figure 1), while the other five *PvARFs* (*PvARF19*, *20*, *39*, *42*, and *43*) are located on unmapped scaffolds. The chromosomal distribution and density of PvARF genes are not uniform. Chr 1, 4, 5, and 7 contain four PvARF gene pairs, respectively. Chr 3 has three pairs, Chr 6 and 9 have only one pair of *PvARFs*, and no gene is located on Chr 2 and 8. Consistent with expectations, 14 gene pairs obviously exist on the two set of chromosomes (Figure 1), while the other 7 gene pairs were putatively located on the chromosomes based on their sequence similarity. The indeed relationships among these *PvARFs* need to be explained by phylogenetic analysis.

### 3.2. Phylogenetic Analysis of Switchgrass ARFs

**Figure 2:**
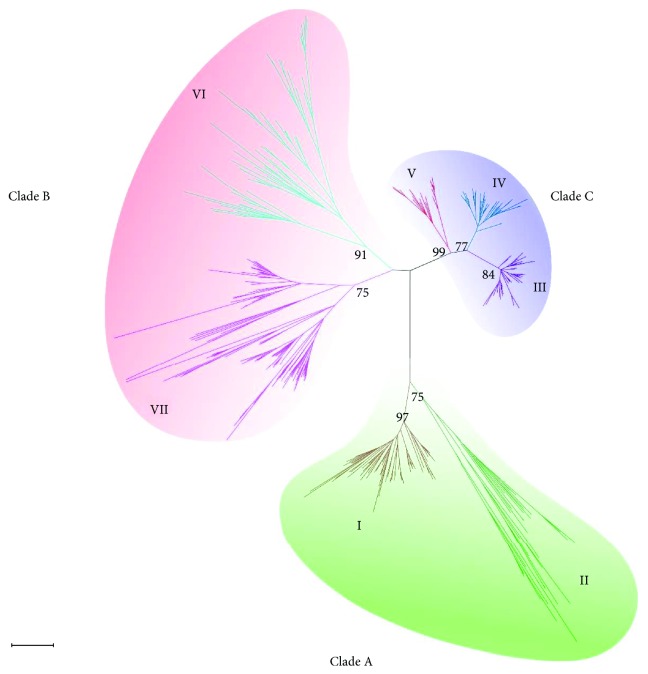
Phylogenetic analysis of the ARF proteins in switchgrass and other plant species. An unrooted neighbor-joining (bootstrap value = 1000) tree was constructed using MEGA5 on the basis of multiple alignments of conserved domain sequences of the ARF proteins from monocot species (switchgrass, foxtail millet, maize, sorghum, rice, and *Brachypodium*) and dicot species (*Arabidopsis*, sweet orange, Chinese cabbage, poplar, *Medicago*, cotton, soybean, tomato, *Grandis*, and grape). And the detailed information was listed in Table S2.

To profoundly characterize the phylogenetic relationships of ARF proteins among switchgrass and other land plants, we selected ARFs from another 15 species, which have public genome database in Phytozome, to construct a phylogenetic tree together with PvARFs. These species include five monocots (foxtail millet, maize, sorghum, *Brachypodium*, and rice) and ten dicots (*Arabidopsis*, sweet orange, Chinese cabbage, poplar, cotton, soybean, *Medicago*, tomato, *Grandis*, and grape). Seven separate clusters of ARF proteins were defined based on the NJ tree topology and bootstrap values (higher than 50%) (Figure 2). As previously reported by Finet et al., three large groups of ARF proteins were classified as clades A, B, and C. In detail, clusters I and II in our study together make up clade C, and clusters III, IV, and V make up clade A. ARF members in these two clades are considered to be more ancient than those in clade B [22], which comprises clusters VI and VII in our study.

Considering that switchgrass is an allotetraploid plant, the gene number of PvARFs in each cluster should be approximately twice than the other monocots, especially in foxtail millet, the most closely relatives to switchgrass among the selected species (Table S2). Cluster VII, which has the largest number (11 out of 47) of PvARFs, contains six foxtail millet ARFs. Cluster I, III, IV, and VI also contain eight switchgrass and four foxtail millet ARFs, and clusters II and V have only two PvARFs, respectively (Table S2). PvARF39 in cluster VII is most closely related to the foxtail millet ARF protein Seita.8G135700.1, which indicates that the paralog of PvARF39 has been lost or mutated gradually during the evolutionary process of switchgrass genome.

In order to comprehensively clarify the evolutionary process of the *PvARFs*, we carried out the tandem repeat duplication analysis based on the chromosomal location and phylogenetic analysis of the *PvARFs*. The results showed that no tandem repeat duplication events were found in *PvARFs*. In addition, we calculated the *Ka*/*Ks* analysis between *PvARFs* and *OsARFs*. Compared with rice ARF genes, 18 pairs of orthologs originated from positive selection (*Ka*/*Ks* ratio was larger than 1), while 6 orthologs showed purifying selection (*Ka*/*Ks* ratio was less than 1) (Table 2).

### 3.3. PvARF Gene Structures and Locations of Conserved Motif

**Figure 3:**
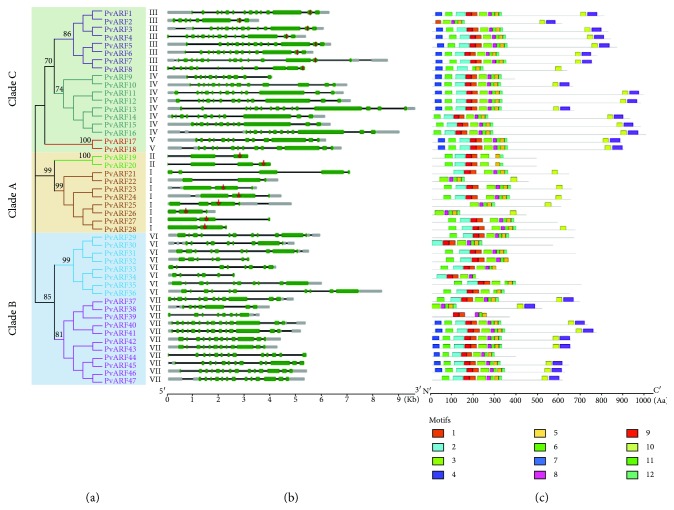
Exon/intron structure of PvARF genes. (a) Phylogenetic tree of PvARF proteins constructed using MEGA5 based on the multiple alignments of full-length amino acids. (b) Exon/intron arrangements of PvARF genes. Exons and introns are represented by green boxes (open reading frame in green, untranslated region (UTR) in gray), and black lines, respectively, and their sizes are indicated by the scale at the bottom. The red vertical bar denotes the targets of Osa-miR167a in PvARF genes; the red arrows denotes the targets of Osa-miR160a in PvARF genes. (c) Schematic representation of conserved motifs in the PvARF proteins predicted by MEME. Each motif is represented by a number in the colored box. The black lines represent nonconserved sequences. Lengths of motifs for each PvARF protein were exhibited proportionally.

**Figure 4:**
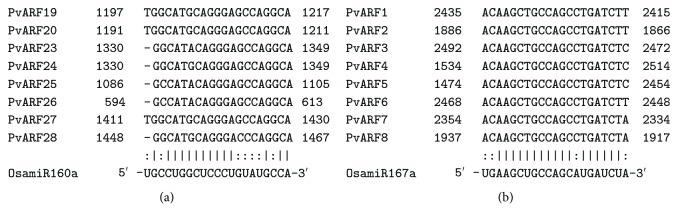
Putative microRNA160 and microRNA167 targeted binding sites of the PvARF genes. (a) Putative microRNA160 targeted binding sites of the PvARF genes. (b) Putative microRNA167 targeted binding sites of the PvARF genes.

To better understand the phylogenetic relationships of the *PvARFs*, the exon/intron arrangements were determined by aligning cDNA sequences to genomic sequences. Another phylogenetic tree was firstly constructed only using switchgrass ARF protein sequences. The PvARF genes were clearly displayed in the form of gene pairs (Figure 3(a)), which confirmed the previous speculation in the chromosomal distribution (Figure 1). All *PvARFs* have introns in the coding sequence (CDS), and the number of introns ranges from 2 to 14 (Table 1, Figure 3(b)). In particular, members belonging to clade C (clusters I and II) contain relatively fewer introns (two to four). In contrast, *PvARFs* in clade A (clusters III, IV, and V) have much more introns (11 to 14), with the exception of PvARF2, which might have lost the exons in N-terminus. The number of introns in clade B (clusters VI and VII) were ranging from 5 to 13. This variability of intron number might be correlated to the multiple functions of clade B *ARFs* in higher plants. Additionally, we further identified the putative microRNA target sites of ARF genes in switchgrass. 16 out of 47 *PvARF*s were found to contain the potential microRNA target sites. Eight *PvARFs* were predicted to be the targets of miR160 and miR167, respectively (Figure 3(b), Figure 4).

Analysis of motif locations in PvARF proteins was performed to explore structural diversity and to predict their functions. A total of 12 conserved motifs were identified using the MEME program (Figure 3(c), Figure S1). The DNA-binding domain (DBD) (motifs 1, 2, and 9 corresponding to Pfam02362) was lost in four members (*PvARF22*, *25*, *25*, and *38*). The ARF domain (motifs 3, 5, 8, and 11 corresponding to Pfam06507) exists in all PvARFs. The AUX/IAA domain (motifs 4 and 10 corresponding to cl03528) has been lost in almost all of the PvARFs in clusters I, II, and VI. These results confirm the phylogenetic relationship between the PvARFs in clades A/C and B and indicate that there has been functional differentiation among PvARFs in different clusters.

### 3.4. Expression Patterns of *PvARFs* in Different Organs of Switchgrass

**Figure 5:**
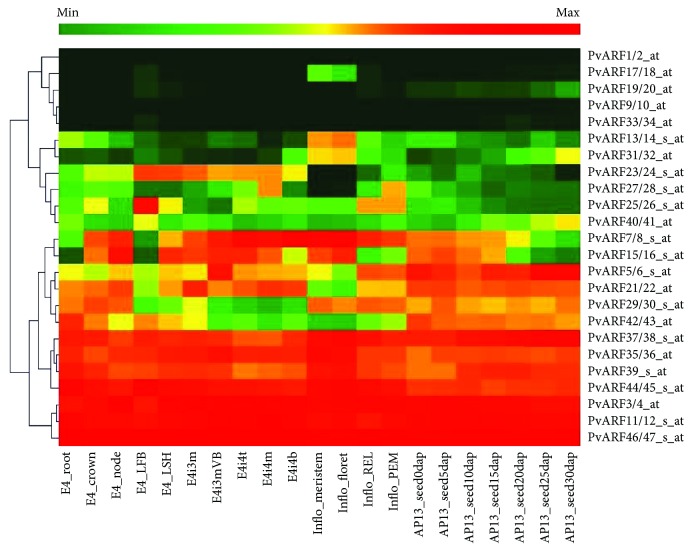
Heatmap of expression profiles of the PvARF gene pairs in different tested tissues. The data was collected from switchgrass gene atlas database. Clustering analysis was carried out using Genesis program (v1.7.6).

To analyze the expression levels of *PvARFs*, we firstly acquired the probesets of the *PvARFs* in switchgrass expression atlas from the public database [38]. The results showed that *PvARFs* were expressed in root, node, internode, leaf, leaf sheath, flower, and seed but with different expression profile of each PvARF gene. For example, *PvARF3*/*4*, 11/12, and *46*/*47* were highly expressed in all tested organs, while *PvARF1*/*2*, *9*/*10*, *19*/*20*, and *33*/*34* were extremely lowly expressed in switchgrass (Figure 5).

**Figure 6:**
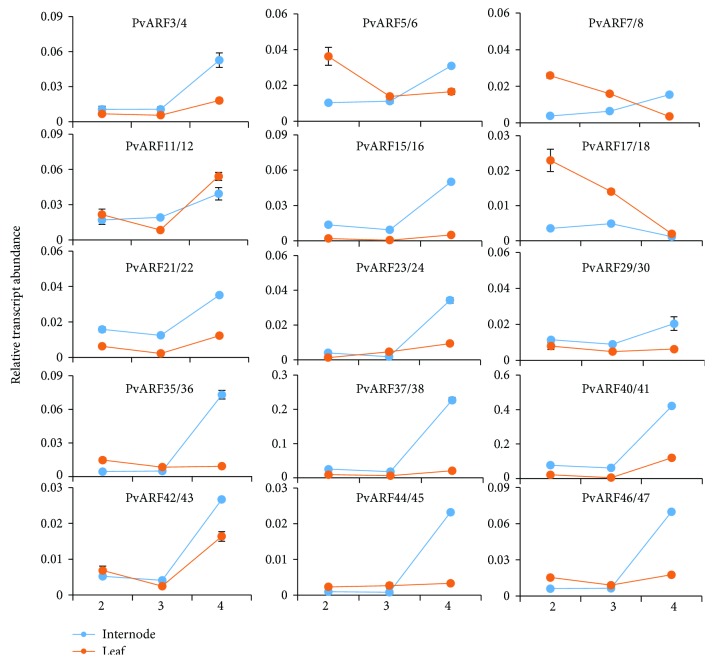
The expression of PvARF genes in the vegetative organs. The expression of PvARF genes in the second internodes (I2), the third internodes (I3), the fourth internodes (I4), the second leaves (L2), the third leaves (L3), and the fourth leaves (L4) of switchgrass. Relative transcript levels are calculated by qRT-PCR. Data are means ± SE of three separate measurements.

Biomass yield is one of the most important criteria used to evaluate the quality of switchgrass. Vegetative organs, especially internodes and leaves, are the primary sources of biomass. Auxin is one of the most important phytohormone, which regulate the plant growth and development. To investigate whether and how PvARFs work on vegetative organs, we selected the second, the third, and the fourth internode and the corresponding leaves at the second reproductive (R2) stage to test the expression profile of the *PvARFs* by qRT-PCR analysis. Eight pairs of PvARF genes and *PvARF39* are not expressed or are extremely lowly expressed in the tested tissues, whereas the other 15 pairs of PvARF genes show substantial expression in internodes and leaves. In internodes, 14 pairs of *PvARFs* have higher expression levels in the upper internode (I4) than the other two internodes (I2 and I3), with one exception (*PvARF17*/*18*) having no obvious difference in the expression level in the three internodes (Figure 6). In leaves, ten pairs of PvARF genes (*PvARF3*/*4*, *15*/*16*, *21*/*22*, *23*/*24*, *29*/*30*, *35*/*36*, *37*/*38*, *40*/*41*, *44*/*45*, and *46*/*47)* show no significant changes in expression level in the three tested leaves. There was lower expression of *PvARF5*/*6*, *7*/*8*, and *17*/*18* in the upper leaf (L4) than in the bottom leaves (L2 or L3), whereas *PvARF11*/*12* and *42*/*43* are more highly expressed in L4 compared to L2, and even lower expression is observed in L3 (Figure 6). These results suggest that the biosynthesis and transport of endogenous auxin in switchgrass might affect the expression profile of PvARF genes, especially in the internode.

### 3.5. Expression Analysis of *PvARFs* in Response to Auxin Treatment

In order to clarify the biofunctions of PvARF proteins, *cis*-acting DNA elements were analyzed using 2 kb promoter sequence of *PvARFs*. The results showed that PvARFs in different clades were putatively involved in specific process. For example, PvARFs in clade A might participate in nodule formation, while clade B genes function on wounding response (Table S3). However, all of the PvARF proteins mostly tended to be involved in plant growth and development, such as phytohormone signaling, abiotic stress response, carbon metabolism, pollen development, and so on (Table S3).

**Figure 7:**
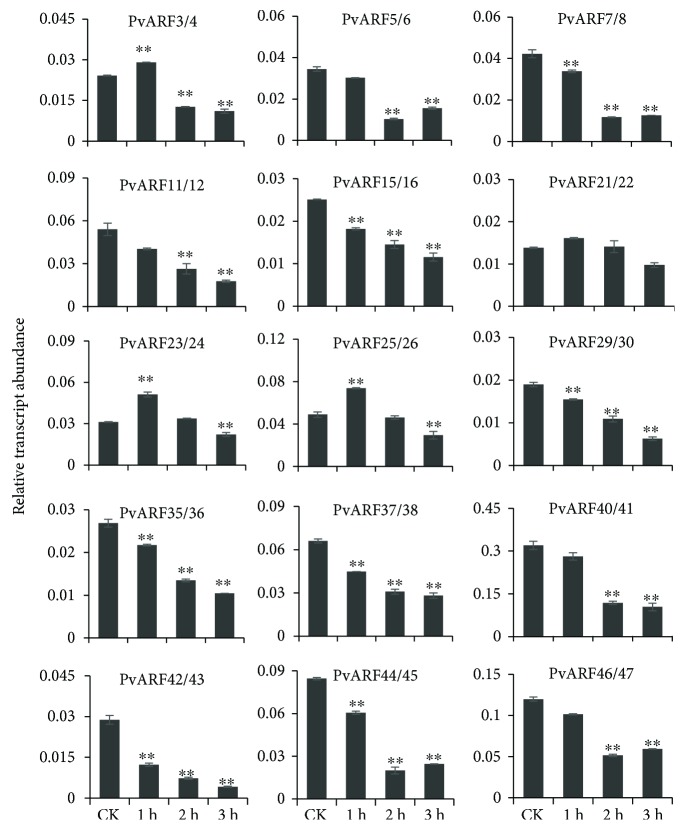
The expression of PvARF genes in response to treatment with 5 *μ*M NAA solution for 1, 2, and 3 hours. Control plants were grown in hormone-free medium. Error bars represented variability of qRT-PCR results from three replicates. Data are means ± SE of three separate measurements. Statistically significant differences were assessed using Student's t-tests (^∗∗^ *P* < 0.01).

As a key component of the auxin signaling pathway, ARF proteins play vital roles in auxin response. It has been reported that auxin induces or represses the expression of some ARF genes in *Arabidopsis* [31], rice [18], and maize [21]. To examine the response of PvARF genes to the exogenous auxin, one-month-old switchgrass seedlings were treated with 5 *μ*M NAA for 0, 1, 2, and 3 hours, and the expression patterns of the *PvARFs* were determined. The qRT-PCR results revealed that auxin repressed the expression of eleven pairs of genes (*PvARF5*/*6*, *7*/*8*, *11*/*12*, *15*/*16*, *29*/*30*, *35*/*36*, *37*/*38*, *40*/*41*, *42*/*43*, *44*/*45*, and *46*/*47*) at all three time points, whereas it induced the expression of three pairs of genes (*PvARF3*/*4*, *23*/*24*, and *25*/*26*) at 1 hour and then reduced at the latter two time points. In contrast, *PvARF21*/*22* expression was not significantly affected by auxin (Figure 7). These results suggest that exogenous auxin could induce or repress the expression of most PvARF genes to regulate switchgrass growth and development.

## 4. Discussion

Extensive studies have shown that *ARFs* play crucial roles in plant growth and developmental processes [10]. However, a systematic analysis of ARF gene members in switchgrass has not been done. In this study, we identified 47 PvARF genes, almost twice than Arabidopsis (23) and rice (25) [18, 31], for the reason of allopolyploidization in switchgrass evolutionary process [41]. Gene structure analysis showed that the number of exons in PvARF genes ranged from 3 to 14, while similar results were found in Arabidopsis [31], rice [18], and tomato [9], which indicates that the plant ARF gene family has highly conserved structures and potentially similar functions across dicotyledonous and monocotyledonous plant species.

Based on phylogenetic analysis, 47 switchgrass ARF genes were assigned to seven separate clusters, which was similar to the previous studies [22]. The number of ARFs of switchgrass in each cluster was about twice than those of five other monocots (maize, rice, sorghum, foxtail millet, and *Brachypodium*) but not consistent with the number of ARFs in ten dicot species (*Arabidopsis*, citrus, Chinese cabbage, poplar, cotton, soybean, *Medicago*, tomato, *Grandis*, and grape), which indicates that differences in the evolution of ARF genes in monocotyledonous and dicotyledonous plants. According to the phylogenetic tree of ARFs from different species, the orthologous relationship was found to dramatically divorce. In cluster V (PvARF17/18-AtARF5-CiARF5-BrARF5-1/5-3-PoptrARF5.1/5.2-GrARF5a/5b-EgrARF5-GmARF40/47-Sl-AF5-VvARF18-ZmARF4/29-OsARF11-Seita.3G028100.1-Sobic.006G255300.1-Bradi5g25157.1), the ratio of orthologous gene number between species is 1 : 1 which suggests that the functions might be well-conserved across species. Orthologous clusters with ratios greater or smaller than 1 : 1 were also found, indicating the functional diversity of ARF genes in switchgrass.

In general, the members of a subgroup are characterized by the presence of conserved domains. According to previous studies, the ARF genes contain several conserved domains, such as motifs 1, 2, and 9 made up the DNA-binding domain, motifs 3, 5, 8, and 11, which correspond to the ARF domain, and motifs 4 and 10, which are located in the C-terminus and correspond to the AUX/IAA super family domain [10]. The high level of conservation of the various motifs among different species indicates that they are involved in similar regulatory pathway. In our study, the C-terminal AUX/IAA super family domain was missing in several gene members, including *PvARF9*, *19*, *20*, *27*, *29–36*, *39*, and *44*, which is consistent with the lack of this domain in *AtARF3*, *13*, and *17* and *MdARF6, 8*, *14*, *17*, *18*, *20*, *21*, and *28* as well as in *SlARF2*, *3*, *7*, and *13* [2, 3, 47]. In addition, PvARF genes which are present in the same clade and possess similar motifs might function redundantly and have similar expression patterns. For example, *PvARF5*/*6* and *7*/*8* which are members of cluster III and *PvARF37*/*38*, *40*/*41*, *42*/*43*, *44*/*45*, and *46*/*47*, which are members of cluster VII exhibit similar expression patterns at the R2 stage.

Switchgrass is an important resource for bioenergy and feedstock materials, and biomass yield is the most important target in molecular breeding of switchgrass. Comprehensive analysis of PvARF gene expression patterns helped us screen for candidate PvARF genes with potentially distinct functions in regulating vegetative organ growth and development. Taken together, 15 pairs of PvARF genes were detected to have high levels of expression in vegetative organs. Similar patterns of expression were also found in tea plants [48], apple [47], and tomato [9]. 13 of the 15 CsARF genes were expressed in root, stem, leaf, flower, and fruit [24]. Eight of the 31 MdARF genes were expressed in stem, leaf, flower, and fruit [46], and 17 SlARF genes were expressed in root, stem, leaf, flower bud, and ovary [9]. In our study, 14 pairs of *PvARFs* were more highly expressed in the I4 than in the I2 and I3, which suggests that these genes might play vital roles during the formation of young stems, and these results are consistent with the reported function of their homologous genes in *Arabidopsis* [26, 49]. However, in leaves, the expression level of most *PvARFs* did not change significantly in different developmental stages. *PvARF11*/*12* is most highly expressed in the fourth leaf, suggesting that these genes may play roles in leaf development like their *Arabidopsis* homologs, *ARF7* and *19* [31]. Of the 47 PvARF genes, 17 were not expressed in the tested tissues, which indicates that they might not function in these organs, or that the functions of these genes may have been lost during evolution. In general, most PvARF genes have different expression profiles in the internodes and leaves, indicating that they might be regulated by the distribution and concentration of endogenous auxin. The in-depth studies will be needed to confirm these results in future.

Because ARF proteins are transcription factors that regulate the expression of auxin response genes, we determined the response of PvARF genes to NAA treatment. The regulation of gene expression in response to auxin has been reported in *Arabidopsis* [3, 31], rice [20, 32, 34], maize [21], tomato [2], *Medicago* [20], and so on. In this study, we found that at least 14 pairs of PvARF genes were responsive to NAA treatment in seedlings but showed diverse expression patterns. Eleven pairs of PvARF genes (*PvARF5*/*6*, *7*/*8*, *11*/*12*, *15*/*16*, *29*/*30*, *35*/*36*, *37*/*38*, *40*/*41*, *42*/*43*, *44*/*45*, and *46*/*47*) were downregulated by exogenous auxin treatment across all time points, indicating that their expression was negatively regulated by NAA, similar to their homologs in rice and maize (*OsARF5*, *14*, and *21* and *ZmARF5* and *18*), of which expression levels decreased marginally in response to auxin [21, 32]. In contrast, the other three pairs of PvARF genes (*PvARF3*/*4*, *23*/*24*, and *25*/*26*) were upregulated by auxin treatment at 1 hour point and then downregulation at later time points, indicating that NAA significantly induced the target gene in a short period of time, as their homologs in *Arabidopsis*, rice, and maize (*AtARF4*, *19*; *OsARF1*, *2*3; and *ZmARF3*, *8*, *13*, *15*, *21*, *27*, and *30*). In brief, expression of these ARF genes increased slightly in response to auxin [21, 31, 32], implying that these genes are potential primary auxin responsive genes. Generally, the expression level of the ARF genes was directly regulated by auxin. Considering that the endogenous auxin concentration is sufficient for plant growth and development, the extra auxin (NAA) applied exogenously might act as inhibitor of auxin response genes in our study, and the further study will be carried out in the future to clarify the mechanism of auxin response in grasses.

## 5. Conclusions

We identified 47 switchgrass ARF genes and established the evolutionary relationship between these genes using phylogenic, gene structure, and conserved protein motif analyses. Expression analyses revealed the potential role of PvARF genes involved in growth and development of switchgrass internodes and leaves and in response to NAA treatment in seedlings. These data provide a solid foundation for future functional characterization of ARF genes and ARF-mediated signal transduction pathway in switchgrass.

## Figures and Tables

**Table 1:** The information of ARF family genes in switchgrass.

Gene name^a^	Gene ID^b^	ORF length (bp)^c^	Deduced polypeptide^d^			Number of intron^e^	Chr^f^	Chr locations^g^
			Length (aa)	MW (kDa)	*pl*			
*PvARF1*	Pavir.Ca02838	2685	894	98.5	5.75	13	3a	47617320–47623645
*PvARF2*	Pavir.Cb00190	2136	711	78	6.02	7	3b	3249145–3252725
*PvARF3*	Pavir.Da00065	2739	912	100.8	5.92	13	4a	1765253–1771355
*PvARF4*	Pavir.Db00366	2781	926	102.1	5.81	13	4b	4362788–4368192
*PvARF5*	Pavir.Aa03303	2721	906	99.5	5.58	13	1a	67423915–67430303
*PvARF6*	Pavir.Ab00451	2715	904	99.5	5.54	13	1b	4856121–4861815
*PvARF7*	Pavir.Ga00205	2502	833	92.8	6.03	13	7a	3171190–3179787
*PvARF8*	Pavir.Gb00274	1992	663	73.7	5.33	11	7b	3192562–3,197923
*PvARF9*	Pavir.Da01885	1296	431	48.1	8.45	10	4a	42434950–42439045
*PvARF10*	Pavir.Db01975	2205	734	81	5.6	13	4b	43526790–43533795
*PvARF11*	Pavir.Fb01896	3282	1093	121.4	6.07	12	6b	48581098–48587963
*PvARF12*	Pavir.Fa00483	3255	1084	120.3	6.14	12	6a	7216236–7223377
*PvARF13*	Pavir.J00164	3549	1182	130.9	6.29	14	contig00149	16339–25992
*PvARF14*	Pavir.Db00232	3231	1076	120	6.28	11	4b	3367149–3373291
*PvARF15*	Pavir.J32718	3246	1081	120.5	6.08	11	contig39521	1–6360
*PvARF16*	Pavir.Ab00366	3174	1057	117.6	6.14	11	1b	4304985–4314018
*PvARF17*	Pavir.Gb00117	2829	942	104.3	5.81	12	7b	1282083–1288213
*PvARF18*	Pavir.Ga00157	2838	945	104.5	5.77	12	7a	2852206–2858929
*PvARF19*	Pavir.J03524	1554	517	56.3	6.24	2	contig04638	11091–14207
*PvARF20*	Pavir.J37640	1548	515	56	6.16	3	contig69503	1–2970
*PvARF21*	Pavir.Ia01695	2040	679	74.4	9.3	4	9a	20465406–20472448
*PvARF22*	Pavir.Ib03238	1374	457	50.1	6.33	2	9b	52838875–52843142
*PvARF23*	Pavir.Da00107	2061	686	74.9	7.04	2	4a	2050051–2053504
*PvARF24*	Pavir.J26437	2067	688	74.8	7.27	2	contig29414	2799–7192
*PvARF25*	Pavir.Aa01271	1836	611	65.4	9.52	3	1a	16431915–16436701
*PvARF26*	Pavir.J17862	1335	444	47.7	8.55	2	contig196091	4–1855
*PvARF27*	Pavir.Gb00635	1851	616	66.1	8.8	2	7b	7512630–7516588
*PvARF28*	Pavir.J24081	2118	705	75.7	7.35	2	contig263498	76–1753
*PvARF29*	Pavir.J08401	2214	737	80.5	7.15	10	contig11657	2523–8428
*PvARF30*	Pavir.Ca00928	1716	571	63.3	9.24	8	3a	10393086–10397991
*PvARF31*	Pavir.Eb02716	2133	710	78.5	6.18	9	5b	59020641–59026106
*PvARF32*	Pavir.J35323	1065	354	39.5	7.62	6	contig52555	409–3563
*PvARF33*	Pavir.J38128	1104	367	40.6	7.98	7	contig73490	486–4550
*PvARF34*	Pavir.J17623	732	243	27.4	8.97	5	contig193925	123–2287
*PvARF35*	Pavir.J17853	2214	737	79.7	7.82	9	contig19600	3358–9328
*PvARF36*	Pavir.Eb03157	2049	682	74.1	6.66	9	5b	65190090–65198384
*PvARF37*	Pavir.Cb00753	2202	733	82	6.26	11	3b	15971362–15976212
*PvARF38*	Pavir.Ca02218	1560	519	58	6.66	6	3a	36368836–36372762
*PvARF39*	Pavir.J22605	1143	380	43.1	9.3	7	contig24640	4150–7685
*PvARF40*	Pavir.Eb03734	2430	809	90.7	6.1	13	5b	71953597–71958902
*PvARF41*	Pavir.Ea03860	2433	810	90.8	6.05	13	5a	61521645–61526769
*PvARF42*	Pavir.Ea00026	2064	687	76.6	5.58	11	5a	658461–662814
*PvARF43*	Pavir.Eb00045	2064	687	76.7	5.62	11	5b	655839–660061
*PvARF44*	Pavir.Ab01961	1440	479	53.7	7.28	12	1b	38024228–38038833
*PvARF45*	Pavir.Aa01676	2136	711	79.3	5.88	12	1a	22183544–22188794
*PvARF46*	Pavir.Gb01617	1986	661	73.5	5.77	13	7b	21374440–21379797
*PvARF47*	Pavir.Ga01750	1923	640	71.1	6.35	12	7a	21881663–21886932

^a^Names referred to the identified PvARF genes in switchgrass in this work. ^b^The alias of each ARF gene in iTAG 2.30 genome annotation. ^c^Length of open reading frame in base pairs. ^d^The number of amino acids, molecular weight (kilodaltons), and isoelectric point of deduced polypeptide calculated by DNASTAR. ^e^The number of intron. ^f,g^Chromosome location from Phytozome (https://phytozome.jgi.doe.gov/).

**Table 2:** *Ka*/*Ks* calculation of ARF genes between switchgrass and rice.

Othologs	*Ka*/*Ks* ratio	Selection pattern
*PvARF1* (*2*) versus *OsARF25*	1.61	Positive selection
*PvARF3* (*4*) versus *OsARF6*	98.11	Positive selection
*PvARF5* (*6*) versus *OsARF17*	2.19	Positive selection
*PvARF8* (*7*) versus *OsARF12*	76.96	Positive selection
*PvARF10* (*9*) versus *OsARF16*	99.00	Positive selection
*PvARF11* (*12*) versus *OsARF21*	0.54	Purifying selection
*PvARF14* (*13*) versus *OsARF19*	7.51	Positive selection
*PvARF15* (*16*) versus *OsARF5*	9.13	Positive selection
*PvARF17* (*18*) versus *OsARF11*	0.44	Purifying selection
*PvARF19* (*20*) versus *OsARF13*	3.91	Positive selection
*PvARF21* (*22*) versus *OsARF22*	14.81	Positive selection
*PvARF23* (*24*) versus *OsARF18*	0.93	Purifying selection
*PvARF25* (*26*) versus *OsARF8*	2.14	Positive selection
*PvARF27* (*28*) versus *OsARF10*	0.06	Purifying selection
*PvARF30* (*29*) versus *OsARF15*	2.81	Positive selection
*PvARF31* (*32*) versus *OsARF2*	3.32	Positive selection
*PvARF33* (*34*) versus *OsARF14*	3.81	Positive selection
*PvARF36* (*35*) versus *OsARF3*	3.99	Positive selection
*PvARF38* (*37*) versus *OsARF24*	0.29	Purifying selection
*PvARF39* versus *OsARF23*	1.22	Positive selection
*PvARF40* (*41*) versus *OsARF4*	0.61	Purifying selection
*PvARF42* (*43*) versus *OsARF1*	1.75	Positive selection
*PvARF45* (*44*) versus *OsARF7*	99.00	Positive selection
*PvARF47* (*46*) versus *OsARF9*	2.38	Positive selection

## Supplementary Materials

Supplementary Materials Figure S1: twelve conserved motifs in PvARF analyzed by MEME search tool. The height of each box represents the specific amino acid conservation in each motif. Table S1: primer sequences used for gene expression analysis by qRT-PCR. Table S2: phylogenetic relationships of ARFs. Table S3: putative *cis*-acting DNA elements in the promoter of PvARF genes.
