# Supplementary figures and images for: Genome-Wide Identification, Phylogeny, and Expression Analysis of ARF Genes Involved in Vegetative Organs Development in Switchgrass
Source: Int J Genomics. 2018 Apr 29;2018:7658910. doi: 10.1155/2018/7658910 (PMC5949158; doi:10.1155/2018/7658910)

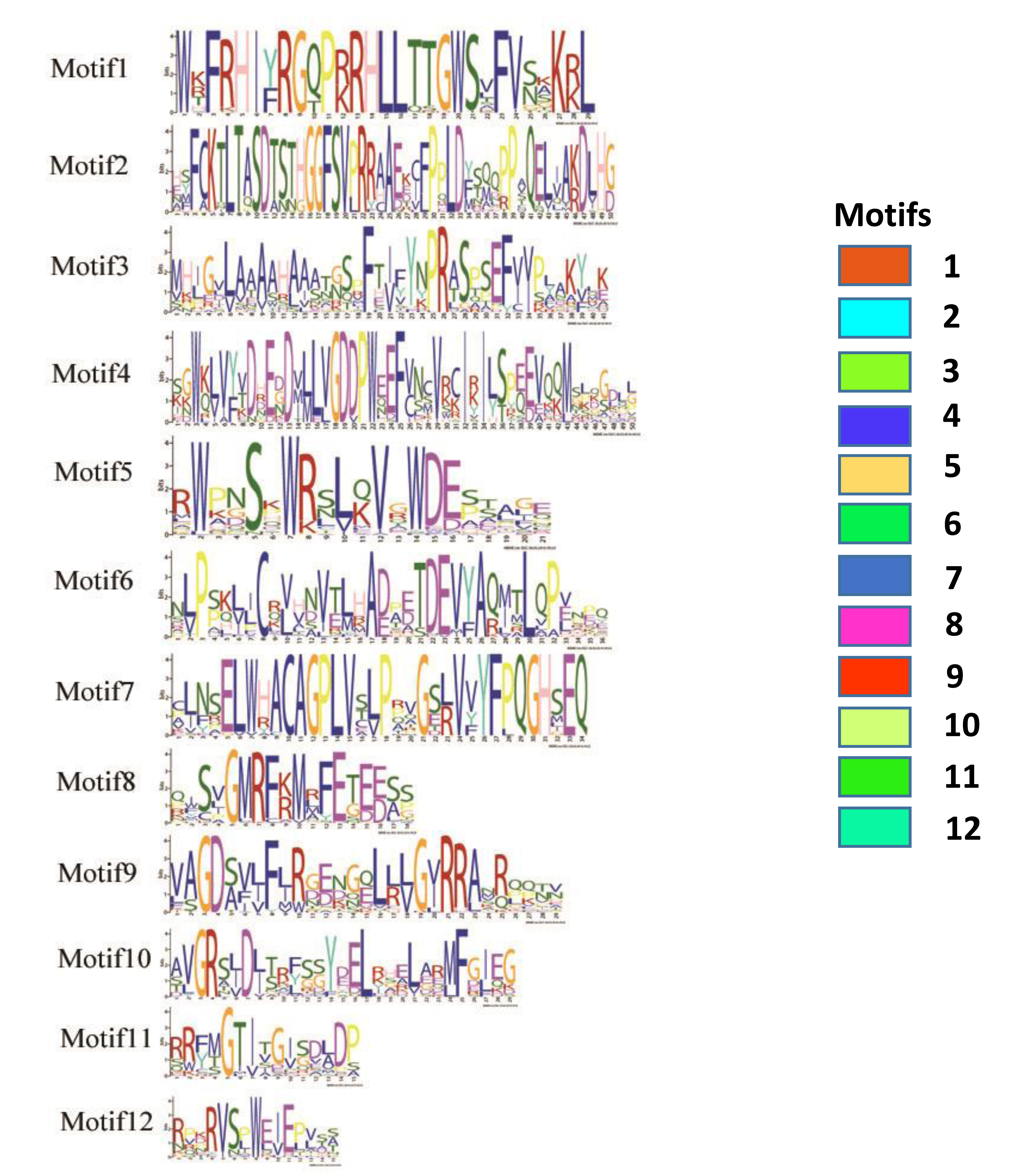

Supplement: Supplementary Materials — Figure S1: twelve conserved motifs in PvARF analyzed by MEME search tool. The height of each box represents the specific amino acid conservation in each motif. Table S1: primer sequences used for gene expression analysis by qRT-PCR. Table S2: phylogenetic relationships of ARFs. Table S3: putative cis-acting DNA elements in the promoter of PvARF genes. [file 7658910.f1.zip › 7658910.f1/Figure S1.jpg]
